# Toxigenic and Antibiotic-Resistant *Bacillus cereus* in Raw Cow Milk from Eastern Cape, South Africa: A Potential Public Health Threat

**DOI:** 10.3390/microorganisms13102253

**Published:** 2025-09-25

**Authors:** Ezekiel Green, Abraham Goodness Ogofure

**Affiliations:** 1 Department of Biotechnology and Food Technology, Faculty of Science, University of Johannesburg, Doornfontein Campus, Johannesburg 2028, South Africa; 2 Microbial Pathogenicity and Molecular Epidemiology Research Group (MPMERG), Department of Biotechnology and Food Technology, Faculty of Science, University of Johannesburg, Doornfontein Campus, Johannesburg 2028, South Africa

**Keywords:** *B. cereus*, Food safety, toxins, refrigeration, contamination of milk, public health

## Abstract

*Bacillus cereus sensu lato* is widespread and causes significant food spoilage that alters the flavour and structure of milk. The present study investigated the prevalence, enterotoxigenic genes, and resistant profiles of *B. cereus* strains isolated from raw milk of *Bos taurus* in South Africa (the Eastern Cape Province). One thousand four hundred samples were obtained from commercial dairy farms and were evaluated for the presence of *B. cereus* using *B. cereus* selective agar, and genomic DNA was isolated from *B. cereus* colonies with specific characteristics. PCR was used to evaluate the presence of enterotoxigenic genes, and antibacterial susceptibility was carried out using the Kirby-Bauer Disc diffusion method. The result revealed that *B. cereus* was detected in 250 raw milk samples. In addition, 67.2% of the isolates grew when incubated at 4 °C. Among the enterotoxigenic genes studied, *ces* showed the highest occurrence (88.8%), but *hblABC* (0%) did not demonstrate amplification from any isolate. Our analysis found two significant patterns (III and V): *nhe*ABC-*ent*FM (27.2% and 24.4%) and the *ces* gene. Total (100%) sensitivity was observed for six of the twelve antibiotics tested, while organisms showed complete resistance to penicillin and rifampicin. This study marks the initial documentation of *B. cereus* and its enterotoxigenic genes in *Bos taurus* raw milk sourced from the Eastern Cape Province, South Africa. Enterotoxin *FM* should be considered the second most crucial enterotoxin, after non-hemolytic enterotoxin, and should be included in the molecular approach used to classify pathogenic *B. cereus* in nutrimental products. These findings underscore the urgent need for public health awareness, particularly in rural communities where raw milk consumption is prevalent. The high prevalence of antibiotic resistance and toxigenic strains of *B. cereus* calls for improved milk pasteurization practices to mitigate the risk of foodborne illness.

## 1. Introduction

*Bacillus cereus sensu lato*, a group of spore-forming, facultative anaerobes, is ubiquitous in water, soil, and food, especially ready-to-eat and dairy products [1,2,3]. Raw milk, widely consumed in rural areas like the Eastern Cape Province, provides a rich medium for psychrotrophic bacteria such as *B. cereus sensu lato*, thereby increasing the risk of foodborne illnesses. The *B. cereus sensu lato group*, which includes *B. anthracis*, *B. thuringiensis*, *B. weihenstephanensis*, *B. mycoides*, *B. toyonensis*, *and B. cereus sensu stricto*, poses a health risk through the consumption of unpasteurized milk, a concern heightened by their ability to form spores, produce toxins, and survive at low temperatures, all of which can compromise milk quality during cold storage [4]. *B. cereus* is often linked to food spoilage and poisoning, and is known to cause illness across all age groups [5]. Infection is associated with vomiting (via cereulide) and diarrhea (via enterotoxins such as *nhe*, *hbl*, *cytK*, and others). A range of virulence enzymes, such as phosphatidyl inositol-specific phospholipase (PI-PLC), enterotoxins (*EntS*), sphingomyelinase (SMase), cereolysin O (*Clo*), and *CytK*, further contribute to its pathogenicity [6]. Molecular studies have linked certain genetic profiles to *B. cereus* virulence, suggesting that horizontal gene transfer may influence its pathogenicity and diversity [7,8,9].

Beyond their notoriety for toxin production, *B. cereus* and related species have also been reported to exhibit potential antibiotic resistance, raising concerns about food safety and public health, even though such resistance is not currently viewed as a major threat. For example, Cui et al. [10], reported the presence of mobile antimicrobial resistance genes in *Bacillus* probiotics, suggesting a theoretical risk to One Health due to the potential transfer of these genes across bacterial populations. Similarly, Navaneethan and Effarizah [11] noted that the emergence of antibiotic resistance in *B. cereus* could contribute to the development of more resistant strains, thereby warranting attention as a possible food safety concern. Furthermore, Bogaerts et al. [12] identified *B. cereus sensu lato* strains in food and feed additives that exhibited both pathogenic potential and resistance to specific antibiotics. While these findings do not establish immediate risks, they underscore the importance of monitoring and mitigating potential hazards associated with *B. cereus* to prevent the dissemination of resistance genes.

*Bacillus cereus* is widely reported in raw milk and dairy products across Europe, Asia, and North America, with its prevalence and toxinogenic potential posing significant public health concerns. Studies in Brazil [13], Thailand [14], South-west China [15], and Greece [16] have demonstrated contamination of raw milk with potential pathogenic *B. cereus* strains, thus emphasizing its ubiquity in dairy products and supply chains.

Despite the global recognition of *Bacillus cereus* as an important cause of foodborne illness, its presence in raw milk from rural commercial dairy farms in South Africa remains poorly characterized. This gap is particularly pronounced in the Eastern Cape Province, where unpasteurized milk consumption is common and cold chain systems are often limited. While sporadic molecular characterization studies on *B. cereus* have been reported from South Africa, there are currently no structured, region-specific epidemiological surveillance studies or comprehensive prevalence data for *B. cereus* in food, animal, or human populations from the Eastern Cape. This contrasts with the extensive surveillance data available from Europe, Asia, and North America, and highlights a significant geographic blind spot in global risk assessments. Our study addresses this gap by providing the first systematic surveillance data on the prevalence and toxigenic potential of *B. cereus* isolated from raw cow milk (*Bos taurus*) in the Nkonkobe District Municipality, Eastern Cape Province, South Africa. By combining prevalence estimates with inferential statistics, diversity indices, and virulence scoring, our study goes beyond descriptive reporting to generate robust baseline data, thus providing a critical One Health foundation for understanding and mitigating *B. cereus*-associated food safety and public health risks in dairy systems.

## 2. Materials and Methods

### 2.1. Sample Collection and Transportation

A total of 1400 raw milk samples were collected aseptically from lactating cows at two large-scale commercial dairy farms in the Eastern Cape Province, South Africa. The two farms are Middledrift Dairy Farm (MDF; 600 samples, with annual production of 1.2 million litres from 600 cows) and Fort Hare Dairy Trust (FHDT; 800 samples, with annual production of 3.6 million litres from 800 cows approximately). They are located in Middledrift and Alice, respectively, within the Nkonkobe Municipality (Amathole District), approximately 90 km northwest of East London and 20 km east of Fort Beaufort. The sample size was chosen to ensure representative coverage of both farms, considering herd size and production output, and to provide sufficient statistical power for downstream analyses.

Prior to sampling, all cows were examined for mastitis. Animals were classified as either clinical (visible signs of mastitis on physical inspection) or subclinical (no external signs but positive California Mastitis Test [CMT]) [17]. Approximately 30–50 mL of milk was aseptically collected from each selected cow into sterile Falcon tubes, following standard hygienic procedures to avoid contamination [18]. Negative controls (sterile Falcon tubes containing sterile PBS) were transported alongside the samples to monitor for environmental or procedural contamination. All samples were kept on ice at 4 °C during transport and processed immediately upon arrival at the University of Fort Hare (UFH) microbiology laboratory.

### 2.2. Bacterial Isolation, Identification and Evaluation of Growth at 4 °C

Bacteriological analyses were performed following standard microbiological protocols: milk aliquots (raw milk samples (10 µL volume)) were cultured on Polymyxin pyruvate Egg-Yolk Mannitol Bromothymol Agar (PEMBA), a diagnostic selective medium for *B. cereus*, and incubated at 37 °C for 48 h. The detection limit of this method is approximately 100 CFU/mL, based on the inoculation volume of 10 µL onto PEMBA. Colonies (blue with white margins, typical of presumptive *B. cereus*) were initially identified based on morphology, Gram and Spore staining, and conventional biochemical tests (catalase, coagulase, oxidase, and sugar fermentation profiles). Furthermore, API 50 CHB (bioMérieux Inc., Marcy-l’Étoile, France) kits were used for the identification of these isolates, and the known reference strain, *B. cereus* ATCC 10876, was employed as a quality control/assurance for the identification of *B. cereus*, as well as for the detection of toxin gene [19]. Additionally, the reference strain, *B. cereus* F4810/72, was used as the quality control strain for the detection of emetic toxin [20]. Negative controls (uninoculated media plates) were incubated alongside the samples to monitor potential laboratory contamination. All phenotypic assays were performed in triplicate to ensure reproducibility.

For the evaluation of bacterial growth at 4 °C, all *B. cereus* isolates identified using phenotypic methods (selective media) were inoculated onto Blood agar base supplemented with laked horse blood (5%). Both the media and supplement were Oxoid (Basingstoke, Hampshire, UK) products, and the incubation was done on 21 d., at 4 °C [21]. *B. cereus* isolates were identified to show or reflect psychotropic tendencies by their abilities to grow at 4 °C within the incubation period. As an additional quality measure, the growth of the positive control strain ATCC 10876 was monitored under the same conditions. Although molecular confirmation (using 16S rRNA sequencing) was not performed for all isolates, the study applied stringent phenotypic characterization to ensure reliable identification in addition to using species-specific primers (Table 1) for confirmation. This limitation is acknowledged and suggests that future studies could integrate molecular typing for higher resolution and confirmation.

### 2.3. Extraction of DNA

Genomic bacterial DNA was extracted using the boiling method described by Chakravorty et al. [22] with little modification. Pure colonies of *B. cereus* were suspended in 200 µL of sterile distilled water (SDW) in an Eppendorf tube. The suspension was heated at 100 °C for 15 min on a Dri-Block DB.2A (Techne, Johannesburg, South Africa) before centrifugation for 10 min at 13500 rpm (Thermo Fisher Scientific, Schwerte, Germany). The DNA template used for analysis was the supernatant from the centrifuged sample in the Eppendorf tube.

### 2.4. Screening for 16S rDNA Gene

Amplification of a 5 µL DNA template was carried out in a reaction mixture (25 µL) which consisted of PCR master mix (12 µL), primers (forward and reverse) PA Forward 5′-AGAGTTTGATCCTGGCT-3′ and PE Reverse 5′-CCTTCAATTCCTTTGAGTTT-3′ (1 µL each), and nuclease-free water (6 µL). The PCR master mix used in the study contained reaction buffer, 0.05 U/μL of *Taq* DNA polymerase, 0.4 mM of each dNTP and 4 mM MgCl_2_. The PCR process involved 35 cycles, each including denaturation at 94 °C for 1 min, followed by annealing for 1 min at 55 °C, and an extension for 1 min at 72 °C. Prior to the cycles, an initial denaturation step at 95 °C was conducted for 5 min, and after the cycles were completed, a final extension step was performed for 7 min at 72 °C [7,23]. The products of PCR were analyzed using 1.8% agarose gel made in 0.5× TAE buffer with ethidium bromide. Approximately 5 µL of the PCR products were combined with 2 µL of 6× gel loading dye and placed onto the gel, which was immersed in 0.5× TAE buffer. A DNA ladder of 100 bp served as the molecular marker for the products of PCR. The gel was run for 50 min at a constant/steady voltage of 100 V/cm. A UV trans-illuminator (Uvitec, Cambridge, UK) was used to visualize the amplicons, and a UV documentation system (Uvitec, Cambridge, UK) was used to photograph the gel. The amplicons were then sent for sequencing at the Central Analytical Laboratory in Cape Town. Sequencing results of representative isolates were compared with GenBank entries using BLAST 2.1.70 to confirm species-level identification.

### 2.5. Antimicrobial Susceptibility Testing

The Kirby–Bauer disk (Oxoid, Basingstoke, UK) diffusion method [24] was employed to evaluate the antibiotic susceptibility profiles of all *B. cereus* samples. Based on prior research [8], we selected 16 prevalent antibiotics. These were ampicillin (10 μg), telithromycin (15 μg), cefepime (30 μg), cephalothin (30 μg), ciprofloxacin (5 μg), vancomycin (30 μg), cefotetan (30 μg), kanamycin (30 μg), amwoxicillin-clavulanic acid (20 μg/10 μg), gentamicin (10 μg), oxacillin (1 μg), rifampin (5 μg), chloramphenicol (30 μg), erythromycin (15 μg), tetracycline (30 μg), and trimethoprim/sulfamethoxazole (1.25 μg/23.75 μg). The antibiotics selected for this study represent those commonly used in both human medicine and veterinary practices, making them relevant for assessing potential cross-resistance between environmental strains and clinical pathogens. The grade was verified using a strain of *Staphylococcus aureus* ATCC 25923. The interpretive groups and zone width breakpoints of 16 antibiotics against *S. aureus* ATCC 25923 were determined following the recommended criteria. *B. cereus* is susceptible to antibiotic tolerance when the zone of inhibition (ZOI) against it is greater than that of the quality control strain, as indicated by a prior study [8]. When the ZOI against *B. cereus* was less than that of the quality control strain, it exhibited resistant antibiotic tolerance. When the ZOI against it was intermediate between resistant and susceptible, it exhibited intermediate antibiotic tolerance.

### 2.6. Detection of gyrB, Toxin and Emetic Toxin Genes in B. cereus

To enhance clarity and conciseness, the key primer sequences, annealing temperatures, and associated references used for the detection of *B. cereus* target genes, including *gyrB*, and genes that are associated with several virulence-associated markers, are summarized in Table 1. All primers were adopted from previously published protocols and validated using appropriate positive control strains. Specifically, *B. cereus* ATCC 10876 was employed as the positive control for all genes except *ces*, for which *B. cereus* F4810/72 served as the reference strain.

**Table 1:** Forward and reverse primers used for PCR in the study.

Target Gene	Forward Primer (5′–3′)	Reverse Primer (5′–3′)	Annealing Temp (°C)	References
*gyrB*	BC Forward 5′-GTTTCTGGTGGTTTACATGG-3′	BC Reverse 5′-TTTTGAGCGATTTAAATGC-3′	57 °C	[19,25]
*nheA*	*nheA* forward 5′-TACGCTAAGGAGGGGCA-3′	*nheA* reverse 5′-GTTTTTATTGCTTCATCGGCT-3′, 51 °C	51 °C	[19,26]
*nheB*	*nheB* forward 5′-CTATCAGCACTTATGGCAG-3	*nheB* reverse 5′-ACTCCTAGCGGTGTTCC-3′	53 °C	[19,26]
*nheC*	*nheC* forward 5′-CGGTAGTGATTGCTGGG-3′	*nheC* reverse 5′-CAGCATTCGTACTTGCCAA-3′	53 °C	[19,26]
*hblA*	*HBLA* forward 5′-GTGCAGATGTTGATGCCGAT-3′	*HBLA* reverse 5′-ATGCCACTGCGTGGACATAT-3′	55 °C	[19,26]
*entFM*	*entFM* forward 5′-ATGAAAAAAGTAATTTGCAGG-3′	*entFM* reverse 5′-TTAGTATGCTTTTGTGTAACC-3′	52 °C	[19,26]
*cytK*	CK-F-1859 5′-ACAGATATCGG(GT)CAAAATGC-3′	CK-R-2668 5′-TCCAACCCAGTT(AT)(GC)CAGTTC-3′	58 °C	[19,26]
*Ces*	*ces* forward 5′-GGT GACACATTATCA TATAAGGTG-3′	reverse 5′GTAAGCGAACCTGTCTGTAAC AACA-3′	54 °C	[20,26]
*L1*	L_1_ forward 5′-AATGGTCATCGGAACTCTAT-3′	L_1_ reverse 5′-CTCGCTGTTCTGCTGTTAAT-3′,	51 °C	[19,26]
*L2*	L_2_ forward 5′-AATCAAGAGCTGTCACGAAT-3′	L_2_ reverse 5′-CACCAATTGACCATGCTAAT-3′	51 °C	[19,26]

All PCR reactions were performed in a 25 µL final volume consisting of 5 µL of DNA template, 12 µL of PCR Master Mix (including 0.05 U/μL Taq DNA polymerase, 0.4 mM of each dNTP, 4 mM MgCl_2_, and reaction buffer), 1 µL each of forward and reverse primers, and 6 µL of nuclease-free water. PCR conditions included initial denaturation, cycling steps (denaturation, annealing, extension), and a final extension tailored to each primer set. The PCR conditions included an initial denaturation for 5 min at 95 °C, followed by 30 cycles of denaturation for 1 min at 94 °C, annealing at a temperature dependent on the primer pair for 45 s, and extension for 2 min at 72 °C. The entire process concluded with a final extension for 7 min at 72 °C. All PCR runs included no-template controls (NTCs) to exclude false positives due to contamination.

### 2.7. Determination of Emetic Toxin and Enterotoxins

The four main toxin genes of *B. cereus*, *cytK1*, *hbl*, *nhe*, and *ces*, were identified as previously outlined [6,9,27]. Enzyme-linked immunosorbent assay (ELISA) tests were employed to identify the generation of *B. cereus* toxins, utilizing monoclonal antibodies that selectively recognize distinct epitopes of each toxin component [28]. Cytotoxicity experiments were conducted on Vero and HEp-2 cells to assess the hazardous potential of *B. cereus* strains resulting from the formation of enterotoxins (*CytK1*, *Hbl*, and *Nhe*) and/or emetic toxin (cereulide) [27,29]. Taken together, these assays provided phenotypic and functional confirmation of virulence potential; however, the absence of whole-genome sequencing or multilocus sequence typing (MLST) remains a limitation that should be addressed in future studies.

### 2.8. Data Analysis and Visualization

Data analysis and visualization were performed using R Studio version 4.4.1, leveraging an integrated suite of specialized packages to ensure accuracy, reproducibility, and clarity in outputs. The analysis pipeline incorporated *ComplexHeatmap*, *tidyr*, *dplyr*, *grid*, and *circlize* [30], which provided robust tools for data manipulation, statistical evaluation, and advanced visualization. Both descriptive and inferential statistical analyses were conducted. Descriptive statistics, including prevalence rates, proportions, and frequency distributions, summarized the occurrence of *Bacillus cereus* isolates and their enterotoxin gene profiles. Inferential analyses were then applied to evaluate the statistical significance of observed patterns. These included Chi-square tests (at 95% confidence interval) for distributional equality, proportion tests for dominant gene patterns, Fisher’s exact tests for pairwise gene associations, and diversity indices (Shannon and Simpson) to quantify gene pattern heterogeneity. Virulence potential was further assessed by scoring the number of toxin genes harboured per isolate, enabling stratification of isolates into low-, moderate-, and high-virulence categories. High-resolution heatmaps were generated with the *ComplexHeatmap* package to visualize the binary presence/absence of toxin genes, supported by hierarchical clustering and custom colour scales for enhanced interpretability. Metadata such as gene identities and positive controls were incorporated using the *rowAnnotation()* function, enriching the biological context. This integrated analytical approach provided a comprehensive framework for both quantitative assessment and visual exploration of complex microbiological datasets. The combination of descriptive trends and inferential testing enabled robust identification of statistically supported patterns in enterotoxin gene distribution, thereby strengthening the epidemiological and One Health implications of the findings.

## 3. Results

### 3.1. Isolation and Toxin Gene Profiling of B. cereus from Raw Milk

From raw milk samples of *Bos taurus* collected in South Africa’s Eastern Cape Province, a total of 250 *B. cereus* strains were isolated using culture-based and molecular approaches. Although the 16S rDNA method remains widely used for bacterial identification, its limitations for precise species-level resolution are well documented. Of the isolates, 168 (67.2%) demonstrated psychrotrophic potential by sustaining growth at 4 °C.

**Figure 1:**
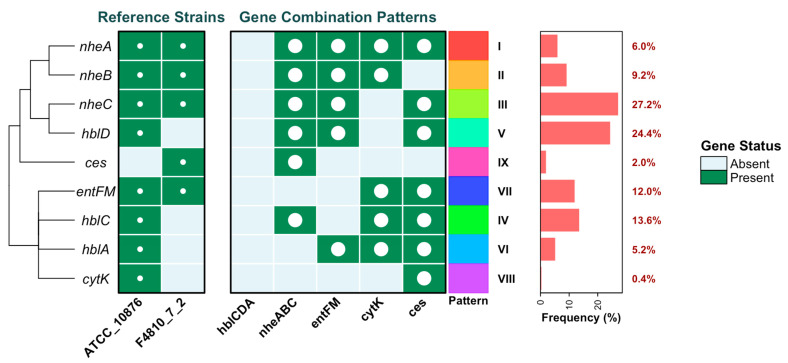
Heatmap of toxin gene distribution in B. cereus isolates from raw milk. Key: *hblCDA* = Hemolysin BL complex (C, D, A subunits); *nheABC* = Non-hemolytic enterotoxin complex (A, B, C subunits); *entFM* = Enterotoxin FM; *cytK* = Cytotoxin K; *ces* = Cereulide synthetase (emetic toxin gene cluster). The left-hand line represents the hierarchical clustering of the virulence genes based on similarity in their distribution patterns, the white dots indicate the presence of a given gene within each combination profile.

The toxin gene distribution among isolates is summarized in Figure 1. Nine toxin genes were investigated (*ces*, *cytK*, *entFM*, *hblA*, *hblD*, *hblC*, *nheA*, *nheB*, and *nheC*). Both reference strains (*B. cereus* ATCC 10876 and F4810/72) expressed the expected gene profiles, although the emetic gene cluster (*ces*) was absent in ATCC 10876 (see Supplementary Table S1). The Chi-square analysis revealed that the distribution of the nine enterotoxin gene patterns among the *B. cereus* isolates was highly uneven (χ^2^ = 158.6, *p* < 0.001). Instead of being equally represented, certain patterns predominated; for example, Pattern III (*nheABC* and *ces* combination) accounted for 27.2% of isolates, and Pattern V (*nheABC* and *cytK*) for 24.4%. In contrast, some profiles, such as Pattern VIII (0.4%) and Pattern IX (2%), were rarely detected. These findings indicate that the occurrence of toxin gene combinations is strongly biased towards particular patterns rather than being randomly distributed across the isolate population. The statistical analyses highlight that *B. cereus* populations in raw milk are not randomly distributed but are instead dominated by a few virulence-rich toxin gene patterns, particularly Pattern III (27.2%) and Pattern V (24.4%), both of which combine *nheABC*, *entFM*, and *ces* genes. The significantly higher prevalence of these patterns compared to others (*p* < 0.001) reflects a non-uniform distribution that raises concern for food safety and public health. The strong genetic associations—such as the co-occurrence of *nheABC* and *entFM* (OR = 10.1, *p* < 0.0001)—indicate evolutionary clustering of virulence determinants, which may enhance pathogenic potential. Despite relatively high overall diversity (Shannon index = 1.866; Simpson index = 0.818), nearly 80% of isolates carried three toxin genes, underscoring a skewed distribution toward highly virulent strains. From a One Health perspective, these findings are crucial: contaminated milk serves as a direct vehicle for zoonotic exposure, with spillover risk to humans, while antimicrobial resistance and toxin gene clustering may further complicate treatment outcomes. Moreover, the persistence of such toxin-rich strains in the food chain exemplifies the interconnectedness of agricultural practices, microbial ecology, and human health, stressing the urgent need for integrated surveillance and risk assessment across the food–environment–human interface.

To evaluate the diversity of *B. cereus* enterotoxin gene patterns, both Shannon and Simpson diversity indices were calculated. The Shannon index (1.866) indicated a moderate-to-high level of pattern diversity, while the Simpson index (0.818) suggested that no single pattern completely dominated the population. This reflects a heterogeneous distribution of virulence profiles among isolates, with multiple toxin gene combinations circulating in raw milk.

**Figure 2:**
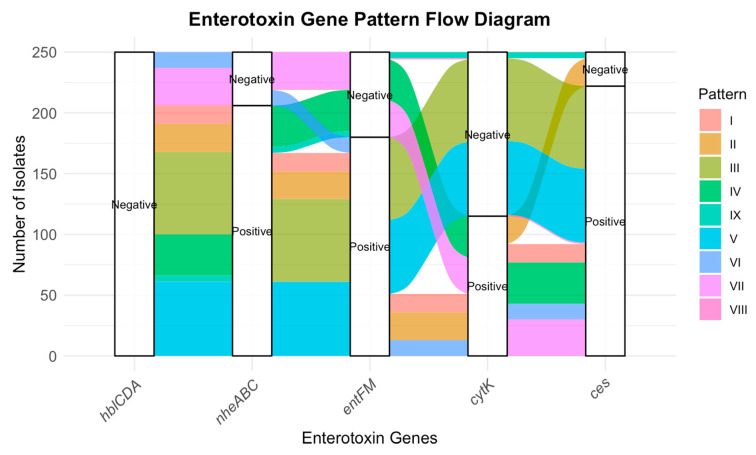
Diversity and virulence potential of *Bacillus cereus* enterotoxin gene patterns in raw milk isolates.

Further analysis of virulence potential, based on the number of toxin genes harboured per isolate (Toxin Score), revealed striking differences in pathogenic capacity (Figure 2). Only 2.4% of isolates carried a single toxin gene (Patterns VIII and IX), while 12% harboured two genes (Pattern VII). The majority of isolates (79.6%) carried three genes simultaneously (Patterns II–VI), representing the most dominant and epidemiologically relevant group. Notably, 6% of isolates (Pattern I) carried all four toxin genes (*nheABC*, *entFM*, *cytK*, and *ces*), underscoring the presence of highly virulent strains with broad pathogenic potential. Together, these results demonstrate that *B. cereus* populations in raw milk are not only genetically diverse but are also heavily skewed toward multi-toxin genotypes, which pose a greater risk to food safety and public health.

**Figure 3:**
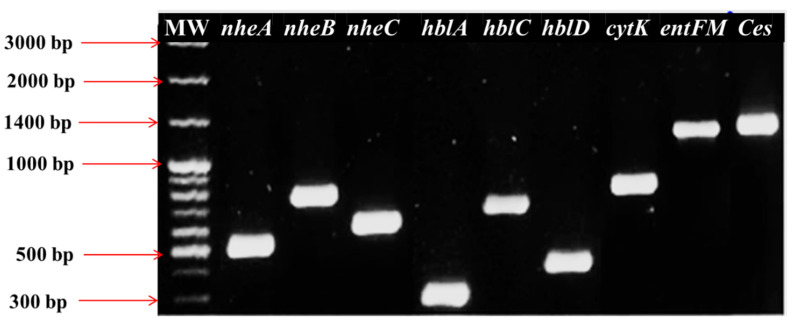
Exemplary PCR results identifying several pathogenic determinant loci in *B. cereus*. Lane MW, 100 bp molecular size DNA marker; lane 1, *nheA*; lane 2, *nheB*; lane 3, *nheC*; lane 4, *hblA*; lane 5, *hblC*; lane 6, *hblD*; lane 7, *cytK*; lane 8, *entFM*; lane 9, *Ces*.

The diversity analysis of enterotoxin gene patterns using Shannon (1.866) and Simpson (0.818) indices shows moderate-to-high diversity, indicating heterogeneous distribution of virulence gene combinations. Virulence potential analysis based on toxin gene scores. Isolates carrying a single toxin gene (2.4%) were rare, while those harboring two genes (12%) or three genes (79.6%) were predominant. A subset of isolates (6%) carried all four toxin genes (*nheABC*, *entFM*, *cytK*, and *ces*), highlighting the presence of highly virulent strains in the population. The circulation of such multi-toxin *B. cereus* genotypes in raw milk raises concerns for food safety and zoonotic transmission, reinforcing the need for a One Health approach that integrates animal, human, and environmental health perspectives in surveillance and risk mitigation. Representative PCR amplification profiles of these virulence determinants are shown in Figure 3, highlighting the presence of multiple toxin genes across the isolates.

### 3.2. Antibiotic Susceptibility Profile of B. cereus from Raw Milk

Antibiotic susceptibility profiles of the isolates (Table 2) show that *B. cereus* was completely resistant to gentamycin and chloramphenicol. The resistance in *B. cereus* may result from genetic mutation or acquisition of resistance genes either through one or a combination of mechanisms such as enzyme inactivation (modification/inactivation of antibiotics), efflux pump (expelling antibiotics to reduce intracellular concentartions), target alteration (changing the binding sites to redue efficiecy) or decreased permeability (modification to cell wall/membrane to prevent entry). Our findings highlight the potential public health risks posed by *B. cereus* in raw milk and underscore the need for continued monitoring of antimicrobial resistance

## 4. Discussion

### 4.1. Isolation and Toxin Gene Profiling of B. cereus from Raw Milk

Previous studies have highlighted challenges in distinguishing certain *Bacillus* species using 16S rDNA analysis [31,32,33,34]. In our study, 16S rDNA similarities amongst species of *Bacillus* isolates from our study ranged from 95 to 98%, consistently showing matches with *B. cereus*, *B. weihenstephanensis*, *B. anthracis*, *B. thuringiensis*, *B. mycoides*, and *B. pseudomycoides*. To improve specificity, we employed species-specific primers targeting *gyrB*, which were highly effective in identifying B. cereus. The sequence variability in the *gyrB* gene (42%) [35], enabled selective amplification of *B. cereus.* In the food industry, *B. cereus* is recognized as a significant foodborne pathogen and spoilage organism, able to resist conventional preservation methods such as pasteurisation and refrigeration. Its resilience in diverse environmental and processing conditions makes it a persistent challenge for dairy safety.

Raw milk provides an ideal growth medium due to its high nutritional content, neutral pH, and water activity. Our findings showed that 168 (67.2%) isolates were able to grow at 4 °C, confirming their psychrotrophic potential. This characteristic poses a food safety risk, particularly for consumers who store raw milk for extended periods. In rural households of the Eastern Cape, where refrigeration infrastructure may be inconsistent, such psychrotrophic growth may increase the likelihood of foodborne illness [36,37,38]. This therefore emphasizes the local importance of our findings, as refrigeration, often considered a protective measure, may not be sufficient to prevent microbial growth.

The detection rate of *B. cereus* in our study was 17.8% of raw milk samples. Importantly, toxin gene screening revealed a high prevalence of virulence determinants: the emetic gene *ces* (88.8%), the non-hemolytic enterotoxin complex *nheABC* (82.4%), and the enterotoxin *entFM* (74%). The cytotoxin *cytK* was also detected in some isolates, though at a lower frequency, while *hblCDA* was completely absent or undetected. These findings demonstrate that local isolates harbour multiple toxin genes, conferring the potential to cause both diarrheal and emetic syndromes. The predominance of *ces* and *nheABC* suggests a strong regional risk profile, with dual capacity for foodborne illness. Comparisons with studies from Asia and Europe reveal both similarities and differences. *B. cereus* strains from food and dairy sources in Korea and China commonly exhibit high prevalence of *entFM* and *nheABC* genes, but the detection of *hbl* genes varies by region and sample [15,39,40,41,42]. This absence may reflect regional variability in genetic profiles. Reports from Europe and North America confirm that B. cereus strains display significant variability in toxin gene combinations (*entFM*, *nheABC*, *hbl*), and that prevalence rates and gene patterns are not uniform across regions [29,43,44,45]. These observations reinforce the need for region-specific surveillance, as South African isolates may differ substantially from global strains.

The identification of multiple toxin genes, particularly the combination of *ces*, *nheABC*, and *entFM* in more than half of the isolates (51.6%), underscores the multifaceted virulence potential of *B. cereus*. This genetic pattern is concerning because it implies that both diarrheal and emetic illnesses may occur simultaneously as symptoms of infection, complicating clinical diagnosis and management. Although diarrheal symptoms are often self-limiting, emetic toxin production can cause acute nausea and vomiting within hours of ingestion, posing serious risks to vulnerable populations such as children, the elderly, and immunocompromised persons. The predominance of *ces* in local isolates is particularly concerning, given its heat-and acid-stable nature [36,37], which allows it to persist or survive even when heated.

Nonetheless, it is important to note that detection of toxin genes does not always equate to functional toxin expression or expression of virulence determinants. Gene expression and toxin production depend on multiple factors, which may include environmental conditions, regulatory pathways, and metabolic states of the organism. Our ELISA assays confirmed the presence of *nheABC*, *entFM*, *cytK*, and *ces* toxins, while *hblCDA* remained undetected, consistent with PCR results. These findings should still be interpreted cautiously, as ELISA can be influenced by assay sensitivity, matrix effects, or partial protein degradation. Future studies incorporating transcriptomics (RT-qPCR) and proteomics will be necessary to validate active expression of these genes under different environmental conditions.

Our results also raise important questions about the psychrotrophic behaviour of local isolates. While growth at 4 °C was clearly observed, studies by Delbrassinne et al. [36], and Jovanovic et al. [46] have shown that cereulide production at strict refrigeration temperatures is often undetectable. This suggests that growth under refrigeration does not always translate into toxin production. Nevertheless, prolonged storage, minor temperature fluctuations, and specific strain variability may still enable minimal toxin accumulation, highlighting that refrigeration alone is not an adequate safeguard. These nuances are critical for interpreting public health risks in households where milk may be stored for more than a week without pasteurisation. The absence of *hblCDA* in our isolates aligns with the findings from Ehling-Schulz et al. [42], Thaenthanee et al. [47] and, more recently, Kim et al. [48], who also reported that emetic strains often lack *hbl* genes. The detection of *cytK* in a subset of the isolates further supports previous evidence that this pore-forming toxin is associated with severe cases of food poisoning, including necrotic enteritis [29,46]. The gene distribution observed in our isolates, dominated by *ces*, *nheABC*, and *entFM*, therefore fits within the globally recognized trends, but with a distinct regional profile marked by the absence of *hbl*. This strengthens the argument that toxin gene patterns in *B. cereus* are geographically variable, reinforcing the need for continuous local monitoring. Another noteworthy outcome of our study was the identification of distinct genetic profiles among *B. cereus* isolates. Two predominant patterns (combinations of *ces*, *nheABC*, and *entFM*) were observed in more than half of the isolates. This mirrors observations by Oliveira et al. [49] but underscores a region-specific risk pattern for South African raw milk. Such patterns highlight the potential for strain-specific adaptation to local environments, perhaps influenced by farming practices, animal health, or milk-handling methods unique to the region.

The public health implications of these findings are considerable, given the widespread consumption of raw milk in the Eastern Cape Province, where pasteurization is not consistently practiced, particularly in rural households. The detection of psychrotrophic *B. cereus* strains harbouring multiple toxin genes indicates a potential food safety risk to consumers. While similar enterotoxin genes have been reported globally, the prevalence patterns and combinations observed here highlight region-specific risks that warrant localized interventions. By combining descriptive and inferential statistical analyses, our study establishes robust baseline evidence for South Africa, enabling targeted risk assessment within the dairy value chain. Practical mitigation strategies should prioritize consumer education on pasteurization, minimizing raw milk storage times, and implementing awareness campaigns in rural communities. Strengthened surveillance systems across South African dairy farms, embedded within a One Health framework linking human, animal, and environmental health, are essential for reducing *B. cereus*-related foodborne illness and monitoring antimicrobial resistance trends.

### 4.2. Antibiotic Susceptibility Profile of B. cereus from Raw Milk

The most prevalent cause of chloramphenicol resistance is the production of chloramphenicol acetyltransferase (CAT), which inactivates chloramphenicol by acetylating it [50]. Efflux pump mechanism and target site modifications also play a role in *B. cereus* resistance to chloramphenicol. The isolates were most sensitive to rifampicin, oxacillin, and cefotetan. Although our findings support a broad picture of antibiotic susceptibility profiles with more resistance than susceptibility, a worrying fact is that some of those antibiotics, such as vancomycin, chloramphenicol, and erythromycin, which were reported in Korea to be effective against *B. cereus* [8,51], show increasing resistance and intermediate resistance in our study.

In total, the resistance of *B. cereus* was found to exceed 80% in 9 out of 16 antibiotics tested in the study, with 5 of the antibiotics showing greater than 90% and 2 with complete resistance (100%). This indicates that over 56.25% of the isolates exhibited multidrug resistance (MDR), defined as resistance to three or more antimicrobial classes, highlighting the emergence of potentially untreatable strains in the food supply. The observed antibiotic resistance, especially to drugs like vancomycin and gentamycin, highlights the potential for resistance genes to persist in the food chain, which may have broader implications for antimicrobial resistance (AMR) dissemination. The antibiotic resistance to first-line drugs, like vancomycin, highlights a potential challenge in managing *Bacillus cereus* infections. This necessitates improved antibiotic stewardship and monitoring of antibiotic use in food production systems. Consumers affected by foodborne illness caused by *B. cereus* may face challenges in treatment due to the organism’s resistance to most antibiotics. Gentamycin, erythromycin and chloramphenicol are alternatively used in treating bacterial infections when first-line drugs fail, but their inefficacy against these isolates suggests limited treatment options. This resistance pattern calls for an urgent review of antibiotic usage in veterinary and human medicine to prevent further resistance development [8,50]. Consumers, especially those in rural areas, should be educated on the importance of pasteurization to reduce the risk of *B. cereus* infection. Public health campaigns could focus on the dangers of raw milk consumption and emphasize safe milk-handling practices.

The antibiotic resistance profiles observed in this study are consistent with global reports of emerging multidrug-resistant *B. cereus* strains. Similar patterns have been documented in China [8,52], Thailand [53], Malaysia [11], Taiwan [54], and Japan [7], where *B. cereus* isolates from food, animals, and the environment exhibit resistance to beta-lactams, tetracyclines and other antibiotics, thus complicating treatment options.

These findings underscore the One Health implications of AMR, as resistance genes can disseminate across ecosystems via contaminated food, shared water sources, or animal waste. Our study reaffirms the necessity of integrated surveillance, judicious antimicrobial use, and cross-sectoral collaboration to mitigate the growing threat of resistant *Bacillus* spp. in both human and animal health domains. While our findings are consistent with reports of multidrug-resistant *B. cereus* in Asia, their relevance lies within the South African context. Rural communities in Eastern Cape frequently consume raw milk without pasteurization, making them particularly vulnerable to infections caused by antibiotic-resistant strains. These findings provide valuable baseline evidence in the South African context, where surveillance data on *B. cereus* in milk remain limited. By applying both descriptive and inferential statistical tools, our study moves beyond simple frequency reporting and establishes statistically supported patterns in toxin gene distribution. Nevertheless, the scope of the work remains region-specific, and broader generalization requires caution. Expanding surveillance to include whole-genome sequencing and comprehensive toxin profiling across multiple provinces would enhance comparability and improve the robustness of epidemiological insights. Such efforts would also allow the integration of molecular epidemiology with public health surveillance, strengthening risk assessment frameworks at both national and global scales.

## 5. Conclusions

This study highlights pressing public health concerns associated with *B. cereus* in raw milk, particularly its toxigenic potential and multidrug resistance. The integration of culture-based methods with both descriptive and inferential statistical analyses enabled the identification of significant patterns in pathogen occurrence and toxin gene distribution. These findings underscore the urgent need for strengthened surveillance systems, improved hygiene practices at the production level, and targeted public awareness campaigns. Promoting safer milk handling and consumption practices, particularly through high-temperature short-time (HTST) pasteurization, remains critical for mitigating foodborne risks. Importantly, our application of inferential statistics lends greater epidemiological weight to the observed trends, enhancing their relevance for local risk assessment. Nonetheless, limitations remain: the study was restricted to two commercial farms in the Eastern Cape and relied primarily on culture-based identification with partial molecular confirmation. These constraints limit the breadth of inference and highlight the need for larger, multi-regional datasets incorporating high-resolution molecular tools. Future research should expand genomic surveillance and statistical modelling to strengthen epidemiological interpretation and to inform evidence-based strategies for mastitis control, food safety management, and antimicrobial resistance monitoring within a One Health framework.

## Figures and Tables

**Table 2:** Antibiotic susceptibility profiles of *B. cereus* isolates from raw milk.

Categories	Antibiotic	Number of Isolates N (%)			
		Concentration (µg)	Resistant	Intermediate	Susceptible
β-lactam antibiotics	Ampicillin	10	160 (64)	90 (36)	0 (0)
	Amoxicillin-clavulanic acid	20/10	190 (76)	17 (7)	43 (17)
	Cephalothin	30	202 (81)	22 (9)	26 (10)
	Cefepime	30	189 (76)	0 (0)	61 (24)
	Cefotetan	30	140 (56)	20 (3)	102 (41)
	Oxacillin	1	28 (11)	0 (0)	222 (89)
Aminoglycosides	Gentamicin	10	250 (100)	0 (0)	0 (0)
	Kanamycin	30	215 (86)	30 (12)	5 (2)
Macrolides	Erythromycin	15	235 (94)	10 (4)	5 (2)
	Telithromycin	15	226 (90)	0 (0)	24 (10)
Glycopeptides	Vancomycin	30	110 (44)	0(0)	140 (56)
Quinolones	Ciprofloxacin	5	237 (95)	0 (0)	13 (5)
Amphenicols	Chloramphenicol	30	250 (100)	0 (0)	0 (0)
Tetracyclines	Tetracycline	30	226 (90)	0 (0)	24 (10)
Rifamycins	Rifampicin	5	6 (2)	0 (0)	244 (98)
Folic acid inhibitors	Trimethoprim-sulfamethoxazole	1.25/23.75	224 (89)	4 (2)	22 (9)

## Data Availability

The original contributions presented in this study are included in the article and Supplementary Materials. Further inquiries can be directed to the corresponding authors.

## Supplementary Materials

The following supporting information can be downloaded at https://www.mdpi.com/article/10.3390/microorganisms13102253/s1, Table S1: Summary of the different enterotoxin genes of *B. cereus* isolates from raw milk.
