# Supplementary material for: Toxigenic and Antibiotic-Resistant Bacillus cereus in Raw Cow Milk from Eastern Cape, South Africa: A Potential Public Health Threat
Source: Microorganisms. 2025 Sep 25;13(10):2253. doi: 10.3390/microorganisms13102253 (PMC12566274; doi:10.3390/microorganisms13102253)
Supplement: Supplementary file 1 [file microorganisms-13-02253-s001.zip › microorganisms-3832417-supplementary.pdf]

Supplementary Materials

Table S1: Summary of the different enterotoxin genes of *B. cereus* isolates from raw milk

	Positive controls		Toxins Genes from Isolates						
Toxin genes	ATCC 10876	F4810/ 72	Pattern	*hblCDA*	*nheABC*	*entFM*	*cytK*	*ces*	Total N (%)
nheA	+	+	I	-	+	+	+	+	15 (6)
nheB	+	+	II	-	+	+	+	-	23 (9.2)
nheC	+	+	III	-	+	+	-	+	68 (27.2)
hblC	+	-	IV	-	+	-	+	+	34 (13.6)
hblD	+	-	V	-	+	+	-	+	61 (24.4)
hblA	+	-	VI	-	-	+	+	+	13 (5.2)
entFM	+	+	VII	-	-	-	+	+	30 (12)
cytK	+	-	VIII	-	-	-	-	+	1 (0.4)
ces	-	+	IX	-	+	-	-	-	5 (2)
Total									250 (100)

Key: *hblCDA: Hemolysin BL complex (components C, D, and A); nheABC: Non-hemolytic enterotoxin complex (components A, B, and C); entFM: Enterotoxin FM; cytK: Cytotoxin K; ces: Cereulide synthetase (emetic toxin gene cluster)*

Library and data frame used in R studio

Final one - Gene Names as First Column with Clustering - ADJUSTED SPACING

```
# Load required packages
library(ComplexHeatmap)
library(tidyr)
library(dplyr)
library(grid)
library(circlize)

# Create the data
toxin_data <- data.frame(
  gene = c("nheA", "nheB", "nheC", "hblC", "hblD", "hblA", "entFM", "cytK", "ces"),
  ATCC_10876 = c(1, 1, 1, 1, 1, 1, 1, 1, 0),
  F4810_7_2 = c(1, 1, 1, 0, 0, 0, 1, 0, 1),
  Pattern = c("I", "II", "III", "IV", "V", "VI", "VII", "VIII", "IX"),
  hblCDA = c(0, 0, 0, 0, 0, 0, 0, 0, 0),
  nheABC = c(1, 1, 1, 1, 1, 0, 0, 0, 1),
  entFM = c(1, 1, 1, 0, 1, 1, 0, 0, 0),
  cytK = c(1, 1, 0, 1, 0, 1, 1, 0, 0),
  ces = c(1, 0, 1, 1, 1, 1, 1, 1, 0),
  percentage = c(6.00, 9.20, 27.20, 13.60, 24.40, 5.20, 12.00, 0.40, 2.00)
)

# Prepare the matrix for controls and gene profiles only
```

```
mat_controls_genes <- as.matrix(toxin_data[, c("ATCC_10876", "F4810_7_2", "hblCDA", "nheABC", "entFM", "cytK", "ces")])

rownames(mat_controls_genes) <- toxin_data$gene


# Create custom color mapping for presence/absence

col_fun <- c("0" = "#E8F4F8", "1" = "#2E8B57")


# Create the comprehensive heatmap with gene names as first column - ADJUSTED SPACING

create_excellent_heatmap <- function() {


 # Gene names annotation (first column) - MUCH CLOSER SPACING

 gene_annotation <- rowAnnotation(

  `Gene Names` = anno_text(

   toxin_data$gene,

   location = unit(0.5, "npc"),

   just = "center",

   gp = gpar(

    fontsize = 12,

    fontface = "italic",

    col = "black",

    family = "Arial"

   ),

   width = unit(1.2, "cm")  # MUCH REDUCED FROM 2.5cm TO 1.2cm

  ),

  annotation_name_gp = gpar(
```

```
    fontface = "bold",
    fontsize = 12,
    col = "black"
  ),
  annotation_name_rot = 0,
  show_legend = FALSE
)

# Pattern annotation
pattern_annotation <- rowAnnotation(
  Pattern = anno_simple(
    toxin_data$Pattern,
    col = structure(rainbow(9, alpha = 0.8), names = unique(toxin_data$Pattern)),
    width = unit(1.5, "cm")
  ),
  `Pattern Label` = anno_text(
    toxin_data$Pattern,
    location = unit(0.5, "npc"),
    just = "center",
    gp = gpar(
      fontsize = 12,
      fontface = "bold",
      col = "#1F4E79",
      family = "Arial"
    ),
```

```
    width = unit(1.5, "cm")
  ),
  annotation_name_gp = gpar(
    fontface = "bold",
    fontsize = 12,
    col = "black"
  ),
  annotation_name_rot = 0,
  show_legend = FALSE
)

# Percentage annotation (last column)
percentage_annotation <- rowAnnotation(
  `Frequency (%)` = anno_text(
    paste0(sprintf("%.1f", toxin_data$percentage), "%"),
    location = unit(0.5, "npc"),
    just = "center",
    gp = gpar(
      fontsize = 12,
      fontface = "bold",
      col = "#8B0000",
      family = "Arial"
    ),
    width = unit(2.5, "cm")
  ),
```

```
  annotation_name_gp = gpar(
    fontface = "bold",
    fontsize = 12,
    col = "black"
  ),
  annotation_name_rot = 0,
  show_legend = FALSE
)

# Create the main heatmap
ht <- Heatmap(
  mat_controls_genes,
  name = "Gene Status",

  # Color scheme
  col = col_fun,

  # Cell appearance
  rect_gp = gpar(col = "white", lwd = 2),
  cell_fun = function(j, i, x, y, width, height, fill) {
    grid.rect(x = x, y = y, width = width, height = height,
              gp = gpar(col = "white", fill = fill, lwd = 1.5))
    if(mat_controls_genes[i, j] == 1) {
      grid.circle(x = x, y = y, r = min(unit.c(width, height)) * 0.3,
                  gp = gpar(fill = "white", col = "white", lwd = 0))
```

```
  }
},

# Row settings with CLUSTERING ENABLED
cluster_rows = TRUE,  # CLUSTERING ENABLED
clustering_distance_rows = "euclidean",
clustering_method_rows = "ward.D2",
show_row_names = FALSE,  # Hide default row names since we have gene annotation
row_dend_side = "left",
row_dend_width = unit(2, "cm"),

# Column settings
cluster_columns = FALSE,
show_column_names = TRUE,
column_names_side = "bottom",
column_names_gp = gpar(
  fontsize = 11,
  fontface = "bold",
  col = "black"
),
column_names_rot = 45,

# Column grouping
column_split = factor(
  c(rep("Reference Strains", 2), rep("Gene Combination Patterns", 5)),
```

```
  levels = c("Reference Strains", "Gene Combination Patterns")
),
column_gap = unit(8, "mm"),
column_title_gp = gpar(
  fontsize = 13,
  fontface = "bold",
  col = "#2F4F4F"
),

# Left annotation - Gene names first
left_annotation = gene_annotation,

# Right annotations - Pattern with colors and Frequency with bar charts - RESTORED
right_annotation = rowAnnotation(
  Pattern = anno_simple(
    toxin_data$Pattern,
    col = structure(rainbow(9, alpha = 0.8), names = unique(toxin_data$Pattern)),
    width = unit(1.2, "cm")
  ),
  `Pattern Label` = anno_text(
    toxin_data$Pattern,
    gp = gpar(fontsize = 10, fontface = "bold", col = "black"),
    width = unit(1, "cm")
  ),
  `Frequency (%)` = anno_barplot(
```

```
    toxin_data$percentage,
    bar_width = 0.8,
    gp = gpar(fill = "#FF6B6B", col = "white", lwd = 0.5),
    width = unit(2.5, "cm"),
    axis = TRUE,
    axis_param = list(gp = gpar(fontsize = 8))
  ),
  `Percent` = anno_text(
    paste0(sprintf("%.1f", toxin_data$percentage), "%"),
    gp = gpar(fontsize = 9, fontface = "bold", col = "#8B0000"),
    width = unit(1.5, "cm")
  ),
  gap = unit(3, "mm"),
  annotation_name_gp = gpar(fontface = "bold", fontsize = 10),
  annotation_name_rot = 0
),

# Legend
heatmap_legend_param = list(
  title = "Gene Status",
  at = c(0, 1),
  labels = c("Absent", "Present"),
  legend_gp = gpar(fill = c("#E8F4F8", "#2E8B57")),
  title_gp = gpar(fontface = "bold", fontsize = 12),
  labels_gp = gpar(fontsize = 11),
```

```
      border = "black",
      legend_height = unit(4, "cm"),
      grid_width = unit(8, "mm")
    ),

    # Overall appearance
    border = TRUE,
    border_gp = gpar(lwd = 2, col = "black"),

    # Dimensions - MEDIUM SIZE
    width = unit(10, "cm"),   # REDUCED from 12cm
    height = unit(8, "cm")    # REDUCED from 10cm
  )

  return(ht)
}

# Create the excellent heatmap
excellent_heatmap <- create_excellent_heatmap()

# Function to draw with perfect layout
draw_perfect_heatmap <- function() {

  # Clear the graphics device
  grid.newpage()
```

```r
# Create main viewport with margins
pushViewport(viewport(
  x = 0.5, y = 0.5,
  width = 0.95, height = 0.9,
  just = c("center", "center")
))

# Add main title
grid.text(
  "Bacillus cereus Toxin Gene Distribution Patterns",
  x = 0.5, y = 0.95,
  gp = gpar(
    fontface = "bold",
    fontsize = 16,
    col = "#2F4F4F",
    family = "Arial"
  ),
  just = "center"
)

# Add subtitle
grid.text(
  "Clustered gene presence/absence patterns with frequency distribution",
  x = 0.5, y = 0.91,
```

```
    gp = gpar(
      fontface = "italic",
      fontsize = 12,
      col = "#555555",
      family = "Arial"
    ),
    just = "center"
  )

  # Draw the heatmap with perfect positioning
  pushViewport(viewport(
    x = 0.5, y = 0.45,
    width = 0.9, height = 0.8,
    just = c("center", "center")
  ))

  draw(excellent_heatmap, newpage = FALSE)

  # Clean up viewports
  popViewport(2)
}

# Alternative simplified version for better compatibility - ADJUSTED SPACING
create_simple_excellent_heatmap <- function() {
```

```r
ht_simple <- Heatmap(
  mat_controls_genes,
  name = "Gene Presence",
  col = col_fun,

  # Appearance
  rect_gp = gpar(col = "white", lwd = 1.5),
  border = TRUE,

  # Clustering - ENABLED
  cluster_rows = TRUE,
  clustering_distance_rows = "euclidean",
  clustering_method_rows = "ward.D2",
  cluster_columns = FALSE,
  show_row_names = FALSE,  # Hide since we have gene annotation

  # Column styling
  column_names_gp = gpar(fontsize = 11, fontface = "bold"),
  column_names_rot = 30,

  # Grouping
  column_split = c(rep("Controls", 2), rep("Gene Profiles", 5)),
  column_gap = unit(5, "mm"),
  column_title_gp = gpar(fontface = "bold", fontsize = 12),
```

```
# Left annotation - Gene names - MUCH CLOSER SPACING
left_annotation = rowAnnotation(
  `Genes` = anno_text(
    toxin_data$gene,
    gp = gpar(fontsize = 11, fontface = "italic", col = "black"),
    width = unit(1.2, "cm")  # MUCH REDUCED FROM 2.5cm TO 1.2cm
  ),
  annotation_name_gp = gpar(fontface = "bold", fontsize = 11)
),

# Right annotations - Pattern with colors and Frequency with bar charts - RESTORED
right_annotation = rowAnnotation(
  Pattern = anno_simple(
    toxin_data$Pattern,
    col = structure(rainbow(9, alpha = 0.8), names = unique(toxin_data$Pattern)),
    width = unit(1.2, "cm")
  ),
  `Pattern Label` = anno_text(
    toxin_data$Pattern,
    gp = gpar(fontsize = 10, fontface = "bold", col = "black"),
    width = unit(1, "cm")
  ),
  `Frequency (%)` = anno_barplot(
    toxin_data$percentage,
    bar_width = 0.8,
```

```
      gp = gpar(fill = "#FF6B6B", col = "white", lwd = 0.5),
      width = unit(2.5, "cm"),
      axis = TRUE,
      axis_param = list(gp = gpar(fontsize = 8))
    ),
    `Percent` = anno_text(
      paste0(sprintf("%.1f", toxin_data$percentage), "%"),
      gp = gpar(fontsize = 9, fontface = "bold", col = "#8B0000"),
      width = unit(1.5, "cm")
    ),
    gap = unit(3, "mm"),
    annotation_name_gp = gpar(fontface = "bold", fontsize = 10),
    annotation_name_rot = 0
  ),

  # Legend
  heatmap_legend_param = list(
    title = "Status",
    at = c(0, 1),
    labels = c("Absent", "Present"),
    title_gp = gpar(fontface = "bold")
  )
)

return(ht_simple)
```

```
}

# Create and display the heatmaps
cat("Creating excellent toxin gene heatmap with clustered genes and closer spacing...\n\n")

# Try the perfect version first
tryCatch({
  draw_perfect_heatmap()
  cat("✓ Perfect clustered heatmap with closer gene spacing created successfully!\n")
}, error = function(e) {
  cat("Trying alternative approach...\n")

  # Use the simple excellent version
  simple_heatmap <- create_simple_excellent_heatmap()

  grid.newpage()
  pushViewport(viewport(width = 0.95, height = 0.9))

  # Title
  grid.text(
    "Bacillus cereus Toxin Gene Distribution Patterns",
    x = 0.5, y = 0.95,
    gp = gpar(fontface = "bold", fontsize = 16)
  )
```

```
  # Draw heatmap
  pushViewport(viewport(x = 0.5, y = 0.45, width = 0.9, height = 0.8))
  draw(simple_heatmap, newpage = FALSE)
  popViewport(2)

  cat("✓ Simple excellent clustered heatmap with closer gene spacing created successfully!\n")
})

# Summary statistics
cat("\n=== PATTERN ANALYSIS SUMMARY ===\n")
cat("Most frequent pattern:", toxin_data$Pattern[which.max(toxin_data$percentage)],
    sprintf("(%.1f%%)", max(toxin_data$percentage)), "\n")
cat("Least frequent pattern:", toxin_data$Pattern[which.min(toxin_data$percentage)],
    sprintf("(%.1f%%)", min(toxin_data$percentage)), "\n")
cat("Total patterns analyzed:", length(toxin_data$Pattern), "\n")
cat("Average frequency:", sprintf("%.1f%%", mean(toxin_data$percentage)), "\n")

cat("\n=== HEATMAP FEATURES ===\n")
cat("✓ Reading order: Gene Names → Controls → Gene Profiles → Pattern → Frequency\n")
cat("✓ Gene names displayed as first column (italicized) - CLOSE TO CONTROLS\n")
cat("✓ Row clustering enabled (Ward.D2 method with Euclidean distance)\n")
cat("✓ Patterns and frequencies on the right side with BAR CHARTS\n")
cat("✓ Exact percentages displayed with bar plots\n")
cat("✓ Professional color scheme and optimized spacing\n")
```

cat("✓ MEDIUM size output (10cm x 8cm) - perfect for publications\n")

cat("✓ Ready for publication in Q1 journal\n") 2, 2), "cm"))
